# Effect of impregnated central venous catheters on thrombosis in paediatric intensive care: Post-hoc analyses of the CATCH trial

**DOI:** 10.1371/journal.pone.0214607

**Published:** 2019-03-28

**Authors:** Yue Wu, Caroline Fraser, Ruth Gilbert, Quen Mok

**Affiliations:** 1 University of Cambridge School of Clinical Medicine, Cambridge, United Kingdom; 2 Population, Policy and Practice Programme, NIHR Biomedical Research Centre, University College London Great Ormond Street Institute of Child Health, London, United Kingdom; 3 Paediatric Intensive Care Unit, Great Ormond Street Hospital for Children, London, United Kingdom; University Magna Graecia of Catanzaro, ITALY

## Abstract

**Purpose:**

The CATheter infections in CHildren (CATCH) trial reported reduced risks of bloodstream infection with antibiotic impregnated compared with heparin-bonded or standard central venous catheters (CVC) in paediatric intensive care. CVC impregnation did not increase the risk of thrombosis which was recorded in 24% of participants. This post-hoc analysis determines the effect of CVC impregnation on the risk of thrombosis leading to CVC removal or swollen limb.

**Methods:**

We analysed patients in the CATCH trial, blind to CVC allocation, to define clinically relevant thrombosis based on the clinical sign most frequently recorded in patients where the CVC was removed because of concerns regarding thrombosis. In post-hoc, three-way comparisons of antibiotic, heparin and standard CVCs, we determined the effect of CVC type on time to clinically relevant thrombosis, using Cox proportional hazards regression.

**Results:**

Of 1409 participants with a successful CVC insertion, the sign most frequently resulting in CVC removal was swollen limb (37.6%; 41/109), with lower rates of removal of CVC following 2 episodes of difficulty withdrawing blood or of flushing to unblock the CVC. In intention to treat analyses (n = 1485), clinically relevant thrombosis, defined by 1 or more record of swollen limb or CVC removal due to concerns about thrombosis, was recorded in 11.9% (58/486) of antibiotic CVCs, 12.1% (60/497) of heparin CVCs, and 10.2% (51/502) of standard CVCs. We found no differences in time to clinically relevant thrombosis according to type of CVC.

**Conclusions:**

We found no evidence for an increased risk of clinically relevant thrombosis in antibiotic impregnated compared to heparin-bonded or standard CVCs in children receiving intensive care.

## Introduction

Central venous catheters (CVCs) are reported to account for around 85% of clinically significant thrombotic events in children [1–3]. Detection of thrombosis is usually based on clinical signs or evidence of CVC malfunction. Ultrasound is not routinely performed, but may be used to diagnose thrombosis before deciding on CVC removal or use of thrombolytic treatment [4]. Reports of the prevalence of clinical signs of thrombosis in children with a CVC as part of their intensive care treatment range from zero to 74% depending on which signs are measured [1, 5, 6]. Signs of intraluminal CVC thrombosis include evidence of the CVC being blocked, such as difficulty withdrawing blood from the CVC, or the need for flushing to unblock the CVC. A swollen limb may indicate extramural venous thrombosis [4, 7]. Both intramural and extramural veno-occlusive thromboses often lead to CVC removal. Replacing CVCs in small, sick children can be very challenging, and reducing the risk of thrombosis is therefore a key consideration when selecting the type of CVC.

The CATCH trial (CATheter infections in CHildren, ISRCTN34884569) reported that antibiotic impregnated CVCs reduced bloodstream infection (BSI) in children compared with standard or heparin-bonded CVCs [8]. It has been shown that both CVC related BSI and CVC related thrombosis have comparable rates as the cause of CVC failure before the completion of treatment [9]. No previous comparisons have been made with regards to the impact of antibiotic impregnated CVCs on the risk of thrombosis in children. Current published studies on CVC related thrombosis in the paediatric setting have been found to be inadequately powered and limited to one centre [2].

The large, multicentred CATCH trial found no significant differences by type of CVC in the time to thrombosis, which was recorded in 24% of trial participants. The CATCH trial defined thrombosis as two or more occurrences of difficulty withdrawing blood, two or more occurrences of flushing to unblock, an occurrence of swollen limb, a positive ultrasound for thrombosis, or CVC removal because of thrombosis [10]. However, a post-hoc sensitivity analysis, which required two reports of swollen limb (prevalence 21%), found a higher risk in antibiotic impregnated CVCs compared to heparin-bonded CVCs (rate ratio for antibiotic vs heparin 1.34; 95% confidence interval: 1.02, 1.77) [10]. This led to concerns that there could be an increased risk of thrombosis with the widespread use of antibiotic impregnated CVCs following the CATCH trial. No other published studies have compared thrombosis risk in antibiotic impregnated CVCs with other types of CVCs in children.

This post-hoc re-analysis of the CATCH trial aims to address concerns that a broad definition of thrombosis may have biased towards a null effect and did not address the effect of antibiotic impregnation on clinically relevant thrombosis that leads to CVC removal. We first conducted a cohort analysis, blind to CVC allocation, to define clinically relevant thrombosis based on the clinical sign most likely to occur in patients who required CVC removal because of concerns of extramural veno-occlusive thrombosis. Second, we conducted post-hoc three-way comparisons of antibiotic, heparin and standard CVCs to determine the effect of type of CVC impregnation on time to clinically relevant thrombosis.

## Methods

### Participants

The Research Ethics Committee for South West England approved the study protocol for the CATCH trial (reference number 09/H0206/69). Participants provided written informed consent to participate in the CATCH study.

The CATCH trial enrolled and obtained consent from 1485 children in 14 paediatric intensive care units (PICUs) in England between December 2010 and November 2012. Details are reported elsewhere [8, 10]. In brief, children admitted to PICU aged less than 16 years who were expected to require a CVC for 3 or more days were randomised to receive an antibiotic (minocycline-rifampicin) impregnated CVC or heparin-bonded CVC or standard CVC (manufactured by Cook Medical Incorporated Bloomington, IN, USA). The primary outcome was time to first bloodstream infection between 48 hours after CVC insertion and 48 hours after CVC removal. A secondary outcome was time to CVC thrombosis. We had access to the trial case report forms (CRFs) but some additional data used in the trial including enhanced death data from the Office for National Statistics (ONS) was not available.

### Cohort analysis to define clinically relevant thrombosis

**Fig 1:**
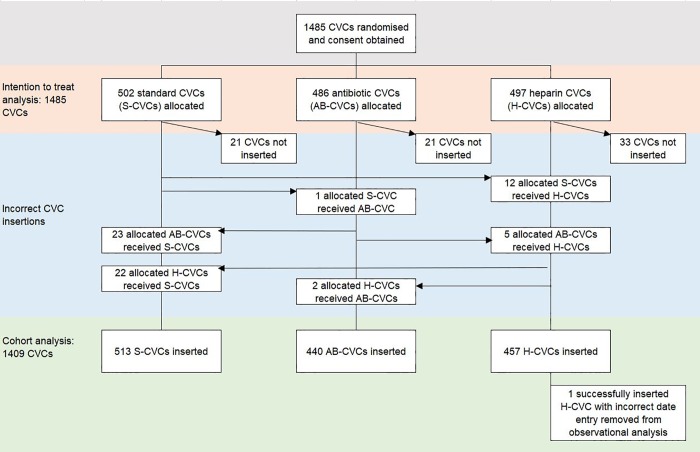
Derivation of study population for the intention to treat and cohort analyses.

The cohort analysis aimed to restrict indicators of thrombosis to those most strongly associated with CVC removal in order to define a combination of signs for clinically relevant thrombosis. The cohort analyses were performed blind to type of CVC impregnation. We restricted analyses to randomised participants who had a successful CVC insertion (n = 1409) (Fig 1) and removed positive ultrasound as this test was rarely performed (10 instances or 0.7%), and in few study centres, usually to confirm thrombosis in patients with a swollen limb. In the CATCH trial swollen limb was recorded by nurses when the swelling persisted beyond the transient swelling noted after insertion attempts. The remaining four clinical criteria for thrombosis were ordered into a descending hierarchy from lowest to highest clinical severity of CVC removal due to thrombosis. For each criterion, we determined the median time from CVC insertion to occurrence of the first clinical sign, to CVC removal because of thrombosis, and to any CVC removal (i.e. CVC duration). For criteria with two or more occurrences of flushing to unblock or difficulty withdrawing blood, we used the date of the second occurrence. If a CVC had more than one clinical sign occurring on the same day, the most severe criterion (at highest risk for CVC removal) was counted as the first event.

To define clinically relevant thrombosis, we determined for which of the three thrombosis criteria (difficulty withdrawing blood twice or more, flushing to unblock twice or more, swollen limb) CVC removal due to thrombosis occurred most frequently.

### Intention to treat analyses of the effect of type of CVC impregnation on clinically relevant thrombosis

We determined the effect of type of CVC impregnation on a composite outcome of clinically relevant thrombosis (CVC removal due to thrombosis or swollen limb) by type of CVC. We analysed groups using intention to treat approach, based on the type of randomised CVC even if patients received no CVC or a different type of CVC was inserted. The primary analysis compared standard CVCs to antibiotic-impregnated CVCs. Secondary analyses compared standard versus antibiotic or heparin, antibiotic versus heparin, and standard versus heparin CVCs. We compared time from randomisation to the first record indicating clinically relevant thrombosis.

### Statistical analysis

In the cohort analyses, we used Cox proportional hazards models to determine whether potential risk factors were significantly associated (p<0.05) with clinically relevant thrombosis compared to the absence of each risk factor. This was performed blind to type of CVC impregnation. The risk factors tested for an association were cardiovascular admission [11], age (<1 year or ≥1 year) [11], CVC insertion in the femoral vein [12–14], anticoagulants received <72 hours before randomisation and systemic infection suspected at time of randomisation [15].

In the intention to treat analyses, we used Cox proportional hazards models to compare the effect of type of CVC impregnation on clinically relevant thrombosis and thrombosis defined by the CATCH trial. We report prevalence of significant risk factors for each CVC type, but we do not adjust for any risk factors as it is a randomised comparison. Schoenfeld residual tests were used to assess whether the proportional hazard assumption was violated in any of the Cox proportional hazard models.

## Results

### Cohort analysis to define clinically relevant thrombosis

Time from CVC insertion to any first clinical sign of thrombosis was shorter for swollen limb (median 3.2 days) than for difficulty withdrawing blood twice (median 4.6 days) and flushing twice to unblock (median 4.8 days) (Table 1). The highest risk of CVC removal because of thrombosis was in participants with a swollen limb (37.6%; 41/109) (S1 Table). In 90.8% (99/109) of these participants, swollen limb was the first presenting sign. Half the participants (49.5%; 54/109) had at least 2 records of swollen limb recorded on separate days. Based on these findings, we defined clinically relevant thrombosis based on CVC removal due to thrombosis or any record of swollen limb. Table 1 shows that clinically relevant thrombosis was recorded in 169 out of 1409 successfully inserted CVCs (12.1%). The only baseline characteristic significantly associated with time to clinically relevant thrombosis (i.e. swollen limb or CVC removal due to thrombosis) was femoral site of CVC insertion (Table 2).

**Table 1:** Cohort analysis to show time to first sign and CVC duration (each of n = 1409 participants counted only once).

Clinical signs of thrombosis (exclusive hierarchy from most severe) ^a^	Frequency^a^ (% of 1409)	Days to any first sign^b^ (IQR)	Duration of CVC ^b^^c^ (IQR)	% with BSI (n/N)
**No clinical signs**	1057 (75.0%)	—	4.0 (2.1, 6.8)	2.0% (21/1057)
**1. Difficulty withdrawing blood (≥ 2 records)**	157 (11.1%)	4.6 (3.6, 7.1)	5.4 (3.6, 7,1)	9.6% (15/157)
**2. Flushing to unblock** **(≥ 2 records)**	26 (1.8%)	4.8 (3.4, 7.4)	6.8 (5.0, 9.9)	3.8% (1/26)
**3. Swollen limb (any)**	68 (4.8%)	3.2 (1.5, 5.2)	5.15 (3.1, 7.8)	2.9% (2/68)
**4. CVC removal due to thrombosis**	101 (7.2%)	3.1 (2.0, 6.1)	3.5 (2.2, 7.3)	3.0% (3/101)
**Total** (any sign)	352 (25.0%)	4.2 (2.6, 6.6)	5.15 (3.0, 7.6)	1.5% (21/1409)
**Total clinically relevant thrombosis** (swollen limb or CVC removal due to thrombosis)	169 (12.1%)	3.2 (1.9, 6.1)	4.2 (2.3, 7.4)	0.4% (5/1409)

Key: IQR = interquartile range, CVC = central venous catheter; BSI = bloodstream infection.

^a^ Frequency given in exclusive hierarchy from most to least severe criterion starting with CVC removal due to thrombosis: i.e. if difficulty withdrawing blood occurred in a participant with CVC removal for thrombosis, then participant is counted in results for CVC removal.

^b^ Days counted from insertion defined as day 0.

^c^ Line duration counted from insertion to CVC removal for any reason in days.

Shading denotes criteria for ‘clinically relevant thrombosis’.

**Table 2:** Baseline characteristics associated with time to clinically relevant thrombosis (crude hazard ratio from Cox proportional regression; cohort analyses n = 1409).

Risk Factor	Frequency n = 1409	Hazard ratio for clinically relevant thrombosis (95% confidence interval)	p-value
Anticoagulants <72 hours prior to randomisation (compared to no anticoagulants)	157 (11.1%)	1.33 (0.88, 2.03)	0.19
Systemic infection suspected at time of randomisation (compared to no systemic infection suspected)	551 (39.1%)	1.14 (0.84, 1.55)	0.41
Femoral insertion (compared to other insertion site)	705 (50.0%)	2.70 (1.88, 3.89)	<0.005
Age ≥1 year (compared to age <1 year)	799 (56.8%)	0.76 (0.55, 1.05)	0.09
Cardiovascular Admission (compared to other reasons for admission)	691 (49.0%)	0.78 (0.57, 1.07)	0.12

### Intention to treat analyses of the effect of type of CVC impregnation on clinically relevant thrombosis

**Fig 2:**
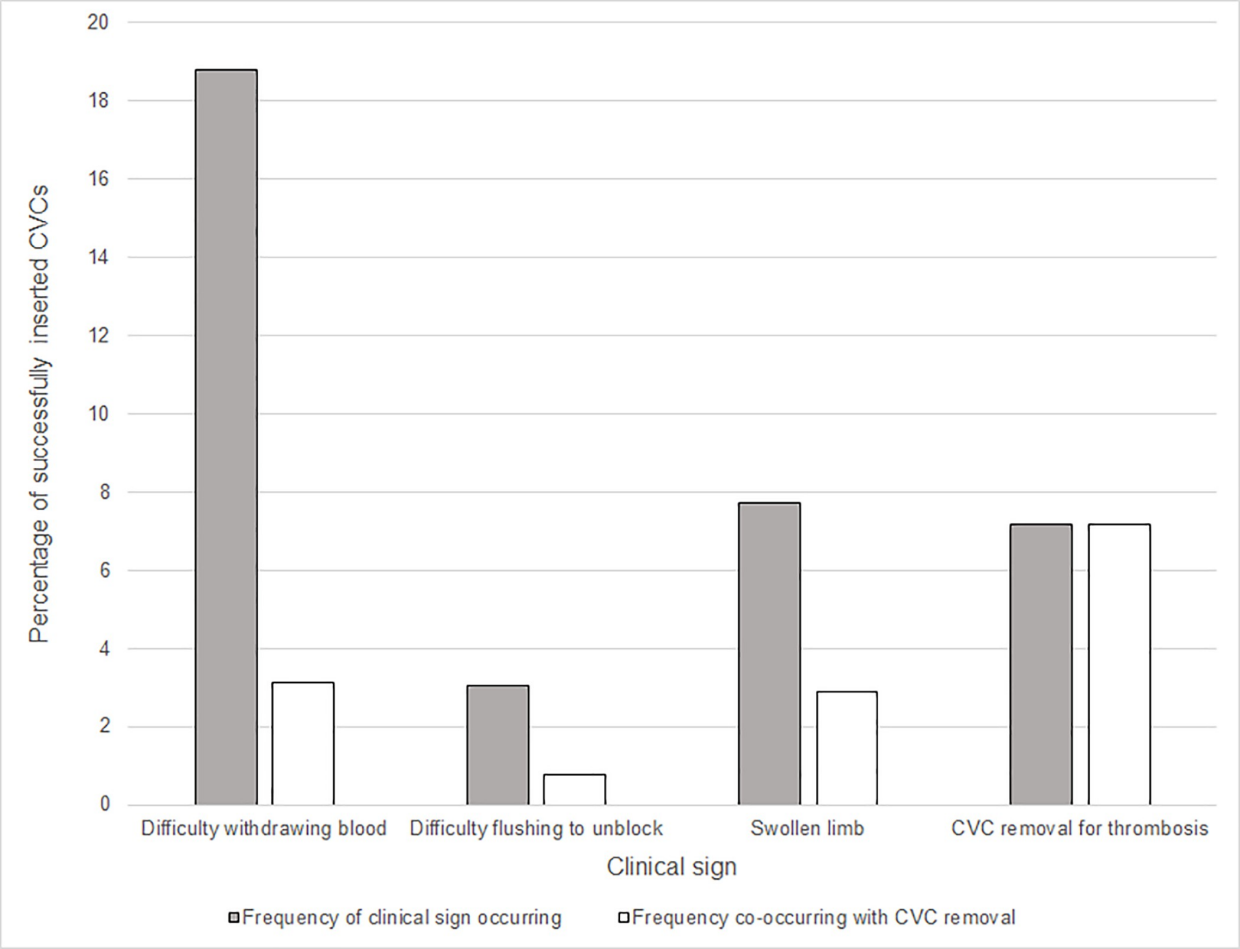
Survival curve showing time to first occurrence of clinically relevant thrombosis by CVC type.

Table 3 shows the proportion of participants according to type of CVC allocation, criteria for thrombosis in the intention to treat analyses of 1485 randomised participants. The distribution of femoral insertion was similar across the three types of CVC with successful insertion: 53% (253/479) for standard CVCs, 53% (235/464) for heparin CVCs and 51% (217/465) for antibiotic CVCs. Distribution of other risk factors are reported in S2 Table. We found no evidence for a significant difference in thrombosis risk according to the type of CVC using clinically relevant thrombosis or the definition used in the original CATCH trial. We found no differences that were significant at the 5% level in comparisons of time to clinically relevant thrombosis between the three types of CVCs (Fig 2; Table 4). Results using the criteria for thrombosis as used in the CATCH trial are presented for comparison. Schoenfeld residual test of proportional-hazards assumption found the assumption was not violated in any of the comparisons (S3 Table).

**Table 3:** The frequency of thrombosis and the cumulative hierarchy of clinical signs by CVC type (intention to treat analyses).

Criteria for thrombosis	Frequency (%)			
	Standard N = 502	Heparin N = 497	Antibiotic N = 486	Total N = 1485
Clinically relevant thrombosis (swollen limb or CVC removal due to thrombosis)	51 (10.2%)	60 (12.1%)	58 (11.9%)	169 (11.4%)
Thrombosis (as defined in original CATCH report)	125 (24.0%)	105 (21.1%)	126 (25.9%)	356 (24.0%)
Results for exclusive hierarchy of signs^a^^b^				
Difficulty withdrawing blood twice	63 (12.5%)	40 (8.0%)	54 (11.1%)	157 (10.6%)
Flushing to unblock twice	10 (2.0%)	3 (0.6%)	13 (2.7%)	26 (1.8%)
Swollen limb	20 (4.0%)	26 (5.2%)	22 (4.5%)	68 (4.6%)
CVC removal due to thrombosis	31 (6.2%)	34 (6.8%)	36 (7.4%)	101 (6.8%)

^a^ Signs ordered in an exclusive hierarchy from most to least severe, starting with CVC removal upwards: ie. the least severe category, ‘difficulty withdrawing blood’ contains no other signs.

^b^ 4 cases of positive ultrasound included in the CATCH report (n = 356) are excluded from the hierarchy of clinical signs (n = 352).

**Table 4:** Comparisons of the effect of type of CVC allocation on time to thrombosis defined as clinically relevant and as pre-specified in the CATCH trial (intention to treat Cox models; n = 1485 randomised CVCs).

Treatment	Number randomised (n = 1485)	Clinically relevant thrombosis^a^			CATCH thrombosis definition ^b^		
		Number experiencing thrombosis (n = 169)	Hazard ratio (95% confidence interval)	p-value	Number experiencing thrombosis (n = 356)	Hazard ratio (95% confidence interval)	p-value
**Baseline comparator: standard**							
**Standard**	502	51			125		
**Antibiotic or Heparin**	983	118	1.26 (0.91 to 1.75)	0.17	231	0.99 (0.80 to 1.23)	0.93
**Antibiotic**	486	58	1.23 (0.85 to 1.80)	0.27	126	1.09 (0.85 to 1.40)	0.48
**Heparin**	497	60	1.28 (0.88 to 1.86)	0.20	105	0.89 (0.69 to 1.16)	0.38
**Baseline comparator: heparin**							
**Antibiotic**	486	58	0.96 (0.67 to 1.37)	0.81	126	1.22 (0.95 to 1.59)	0.12

^a^ Clinically relevant thrombosis definition–CVC removal due to thrombosis or any record of swollen limb

^b^ CATCH thrombosis definition–CVC removal due to thrombosis; any record of swollen limb; any positive ultrasound record for thrombosis; two or more occurrences of flushing to unblock or two or more occurrences of difficulty withdrawing blood

## Discussion

In view of the evidence from the CATCH trial that antibiotic CVCs reduce blood stream infection rates and are cost effective during hospitalisation [10], there were concerns that the widespread introduction and use of antibiotic-impregnated CVCs would increase the risk of thrombosis. In these post-hoc analyses we defined clinically relevant thrombosis as CVC removal due to thrombosis, or one or more occurrence of swollen limb, which was an early sign and was followed by CVC removal in over a third of patients. Clinically relevant thrombosis occurred in 12% of participants, less than half of the 25% identified with thrombosis using the criteria pre-specified in the original CATCH trial analysis. Intention to treat analyses showed no evidence of an association between the type of CVC and thrombosis defined using criteria for clinically relevant thrombosis or as used in the original CATCH trial.

Our findings are consistent with the findings of 2 previous randomised trials comparing antibiotic impregnated and standard CVCs in adults, and 2 trials comparing heparin-bonded with standard CVCs in children, showing no evidence of effect on thrombosis [16, 17]. Our inclusion of swollen limb in the criterion for clinically relevant thrombosis is supported by one cohort study of 389 children, which reported swollen limb to be strongly associated with venous thromboembolism confirmed by radiology records [18]. Consistent with our findings, this cohort study found that signs of difficulty withdrawing blood and flushing to unblock the CVC were much more common but had a lower risk of CVC removal, possibly because the CVC remains functional. CVC malfunction due to other causes including intraluminal thrombus, mechanical occlusion, precipitation of drug or parenteral nutrition or CVC malposition is often partial [7, 19].

We found that inserting CVCs into the femoral vein was significantly associated with clinically relevant thrombosis, as reported in other studies of children [12, 14].

Cardiovascular disease has been previously identified as a risk factor for thrombosis although this was not significantly associated in our study [1, 20]. This may be because we used a relatively non-specific indicator of cardiovascular disease (reason for admission). Although age <1 year has also previously been identified as a risk factor for thrombosis [10], we found no significant association with younger age in our study. This may be due to the insertion of CVCs in the upper venous system in around 50% of the patients in our cohort.

The strength of our study is that it is the first large randomised trial of antibiotic impregnated CVCs in children in intensive care that prospectively measured risk factors for thrombosis, allowing analysis of different criteria for thrombosis. Our findings of no evidence of an effect of CVC type on clinically relevant thrombosis is likely to be generalizable as CATCH was a large pragmatic trial that closely resembled the population of children receiving paediatric intensive care in England. One limitation of the CATCH study was that the staff were not fully blinded to CVC allocation, as the antibiotic CVC was identifiable at insertion or removal by the brown colour of the part of CVC inserted into the patient. However this is unlikely to have affected recording of signs of thrombosis as the CVC types were indistinguishable whilst the CVC was in situ. Our Hazard ratios differ slightly to those reported for the CATCH trial due to differences in censoring dates [8].

### Implications

Swollen limb is an early sign of clinically relevant thrombosis resulting in CVC removal. We found no evidence for an increased risk of clinically relevant thrombosis with antibiotic impregnated CVCs compared to heparin-bonded or standard CVCs.

## Supporting information

S1 Table Frequency of clinical signs overall and co-occurrence with CVC removal due to thrombosis. (DOCX)

S2 Table Thrombosis risk factors at baseline by CVC type. (DOCX)

S3 Table Results from Schoenfeld residuals test for proportional hazards assumption. (DOCX)

S1 File Thrombosis minimal data Excel spreadsheet. (XLSX)
