# Supplementary material for: Effect of impregnated central venous catheters on thrombosis in paediatric intensive care: Post-hoc analyses of the CATCH trial
Source: PLoS One. 2019 Mar 28;14(3):e0214607. doi: 10.1371/journal.pone.0214607 (PMC6438638; doi:10.1371/journal.pone.0214607)
Supplement: S1 Table — (DOCX) [file pone.0214607.s001.docx]

**S1 Table**

**Frequency of clinical signs overall and co-occurrence with CVC removal due to thrombosis**

| **Clinical criteria for thrombosis** | **Frequency**  **(% of 1409)** | **Frequency co-occurring with CVC removal due to thrombosis**  **(row %)** |
| --- | --- | --- |
| **Difficulty withdrawing blood twice (≥ 2 records)** | 265 (18.8) | 44 (16.6) |
| **Flushing twice (≥ 2 records)** | 43 (3.1) | 11 (25.6) |
| **Swollen limb (any)** | 109 (7.7) | 41 (37.6) |
| **CVC removal due to thrombosis** | 101 (7.2) | 101 (100.0) |
