# Supplementary material for: Effect of impregnated central venous catheters on thrombosis in paediatric intensive care: Post-hoc analyses of the CATCH trial
Source: PLoS One. 2019 Mar 28;14(3):e0214607. doi: 10.1371/journal.pone.0214607 (PMC6438638; doi:10.1371/journal.pone.0214607)
Supplement: S2 Table — (DOCX) [file pone.0214607.s002.docx]

**S2 Table**

**Thrombosis risk factors at baseline by CVC type**

|  | **Standard CVC (n=502)** | **Antibiotic-impregnated CVC (n=486)** | **Heparin-impregnated CVC (n=497)** |
| --- | --- | --- | --- |
| **Age** |  |  |  |
| <1 year | 282 (56%) | 282 (58%) | 291 (59%) |
| ≥1 year | 220 (44%) | 204 (42%) | 206 (41%) |
| **Reason for admission*** |  |  |  |
| Cardiovascular | 235 (49%) | 233 (51%) | 250 (50%) |
| Other | 244 (51%) | 153 (49%) | 247 (50%) |
| **Insertion site** ⱡ |  |  |  |
| Femoral vein | 253 (53%) | 217 (47%) | 235 (51%) |
| Other | 228 (47%) | 248 (53%) | 229 (49%) |
| **Anticoagulants <72 hours before randomisation** |  |  |  |
| Yes | 50 (10%) | 59 (12%) | 61 (12%) |
| No | 452 (90%) | 427 (88%) | 436 (88%) |
| **Systemic infection suspected at randomisation** |  |  |  |
| Yes | 214 (43%) | 181 (37%) | 199 (40%) |
| No | 288 (57%) | 305 (63%) | 298 (60%) |

*n=429 for standard CVCs, n=456 for antibiotic-impregnated CVCs, n=497 for heparin-impregnated CVCs. ⱡn=481 for standard CVCs, n=465 for antibiotic-impregnated CVCsand n=464 for heparin bonded CVCs
