# Supplementary material for: Effect of impregnated central venous catheters on thrombosis in paediatric intensive care: Post-hoc analyses of the CATCH trial
Source: PLoS One. 2019 Mar 28;14(3):e0214607. doi: 10.1371/journal.pone.0214607 (PMC6438638; doi:10.1371/journal.pone.0214607)
Supplement: S3 Table — (DOCX) [file pone.0214607.s003.docx]

**S3 Table**

**Results from Schoenfeld residuals test for proportional hazards assumption**

| **Comparison** | **Clinically relevant thrombosis** | | | **CATCH thrombosis** | | |
| --- | --- | --- | --- | --- | --- | --- |
|  | **Chi squared** | **degrees of freedom** | **p-value** | **Chi squared** | **degrees of freedom** | **p-value** |
| **Baseline comparator: standard** | | | | | | |
| **Antibiotic or heparin** | 0.73 | 1 | 0.39 | 0.3 | 1 | 0.58 |
| **Antibiotic** | 1.22 | 1 | 0.27 | 0.11 | 1 | 0.74 |
| **Heparin** | 0.16 | 1 | 0.69 | 0.35 | 1 | 0.55 |
| **Baseline comparator: heparin** | | | | | | |
| **Antibiotic** | 0.52 | 1 | 0.47 | 0.21 | 1 | 0.65 |
